# The Benefit and Safety of Aspirin for Primary Prevention of Ischemic Stroke: A Meta-Analysis of Randomized Trials

**DOI:** 10.3389/fphar.2016.00440

**Published:** 2016-11-18

**Authors:** Hong Lei, Qian Gao, Shan-Rong Liu, Jian Xu

**Affiliations:** ^1^Institute for Drug and Instrument Control of Beijing Military Area CommandBeijing, China; ^2^Department of Laboratory Medicine, Changhai Hospital, Second Military Medical UniversityShanghai, China

**Keywords:** aspirin, stroke, safety, gastrointestinal bleeds, prevention

## Abstract

**Background:** Although aspirin is effective in the secondary prevention of stroke among men and women, its use in primary prevention remains controversial. We conducted a meta-analysis of randomized trials to evaluate the benefit and safety of aspirin for the primary prevention of ischemic stroke.

**Methods:** We searched three electronic databases (Medline, the Cochrane Central Register of Controlled Trials, and Embase) for articles published before August 1st, 2016. Randomized trials reporting the effect of aspirin on the primary prevention of ischemic stroke and its side effects (hemorrhagic stroke and severe gastrointestinal bleeding) were included. We used a fixed-effect model to quantify the effect of aspirin on the primary prevention of stroke when the heterogeneity was low, or else applied the random-effect model.

**Results:** Fourteen randomized trials were included. Overall, aspirin use was associated with a decreased risk of ischemic stroke compared with non-aspirin use (OR: 0.83, 95% CI: 0.74–0.93, *P* = 0.45). In subgroup analyses, the effect of aspirin on ischemic stroke in apparently healthy adults remained significant (OR: 0.83, 95% confidence interval: 0.74–0.94, *I*^2^ = 22%, *P* = 0.28); while in patients with cardiovascular diseases there was no difference in the risk of ischemic stroke between aspirin and non-aspirin groups (OR: 0.75, 95% confidence interval: 0.44–1.29, *P* = 0.46). As for adverse effects, the prophylactic use of aspirin potentially increased the risk of serious bleeding events in a population of apparently healthy individuals and in patients with previous cardiovascular diseases.

**Conclusion:** This meta-analysis of randomized trials indicated that both the apparently healthy adults and patients with cardiovascular diseases will derive little protective benefit from aspirin considering the increased risk of severe bleeding events.

## Introduction

The use of aspirin for the prevention of stroke is already fairly widely spread in many communities. Although it is effective in the secondary prevention of stroke among men and women, its use in primary prevention remains controversial (Cuzick et al., 2015; Kuznar and Uchiyama, 2015; Kwok et al., 2015). The most recent AHA (American Heart Association) guideline for the primary prevention of cardiovascular disease and stroke recommends the use of aspirin in persons whose 10-year risk for coronary heart disease is 6–10% to improve the likelihood of a positive balance of coronary risk reduction over bleeding and hemorrhagic stroke caused by aspirin. It also suggests aspirin is not useful for preventing a first stroke in person at low risk (Goldstein et al., 2011). Thus, the use of aspirin for cardiovascular (including but not specific to stroke) prophylaxis is recommended for persons whose risk is sufficiently high for the benefits to outweigh the risks associated with treatment. Since these recommendations, each patient should undergo an assessment of stroke risk to determine who might benefit from therapeutic interventions. Although independent stroke predictors such as age, hypertension, and diabetes mellitus are identified, an ideal stroke risk-assessment tool that is generally applicable, simple and widely accepted does not exist, making the use of aspirin for preventing a first stroke in persons at elevated risk extremely ambiguous (Chobanian et al., 2003; Kissela et al., 2005; Sturgeon et al., 2007).

Recent systematic analysis of the outcomes from the nine randomized controlled trials confirmed that aspirin had no statistically significant effect on stroke (Bartolucci et al., 2011). However, the included primary prevention trials compared the incidence of all strokes. The reduction in occlusive events might be offset by any increase in cerebral bleeds since the prophylactic use of aspirin could potentially increase the risk of hemorrhagic stroke (Sato et al., 2006; Paciaroni et al., 2007). Under that circumstance, harm from cerebral bleeds might outweigh the benefit from aspirin. Meanwhile, there was another possible explanation for the conclusion that aspirin had no statistically significant effect on stroke: aspirin failed to protect ischemic stroke and didn't increase the risk of hemorrhagic stroke as well. The effects of aspirin on ischemic stroke and hemorrhagic stroke needed to be evaluated respectively. The reasons were as follows: if aspirin had no significant effects on both strokes, then it was not recommended for prevention of stroke but could still be prescribed to prevent other cardiovascular events such as coronary heart disease. If aspirin increased the risk of major bleeds (although it decreased the incidence of ischemic stroke), then it would be avoided for the prevention of stroke and should be prescribed with great caution to prevent other cardiovascular diseases.

In addition, it has been found that aspirin has differential effects in distinct populations. The previous meta-analysis investigating the effect of low-dose aspirin on the primary prevention of stroke included nine clinical trials, which enrolled both patients with previous cardiovascular events and apparently healthy volunteers. Numerous studies found the overall benefit of aspirin is confined to those with low pressures while men with pressures of more than 145 mmHg will derive little cardiovascular protective benefit from aspirin (Meade and Brennan, 2000). In patients with type 2 diabetes, low-dose aspirin as primary prevention did not reduce the risk of ischemic stroke (Ogawa et al., 2008). The results of US trial raised the possibility that aspirin may have been more effective in those aged 50 years or more and when cholesterol concentrations were low rather than high (Steering Committee of the Physicians' Health Study Research Group, 1989). For healthy individuals, aspirin may have only a modest effect as a primary prevention of stroke (Baigent et al., 2009). Thus, an important concern in the previous meta-analysis was confounding by factors associated with the stroke types and type of people studied. In this meta-analysis, we aimed to compare the effects of aspirin on the incidence of ischemic stroke and hemorrhagic stroke respectively, and hope to identify a higher-risk group who might derive substantial benefit from aspirin therapy.

## Methods

### Literature search and study selection

We attempted to identify all randomized controlled trials that evaluated aspirin treatment as compared with a control (placebo or no aspirin), that included data on the incidence of stroke and adverse events, and that were published on or before August 1st, 2016. We conducted searches of Medline, the Cochrane Central Register of Controlled Trials, and Embase. There were no language limitations for the initial search. Cohort studies and case-control studies were excluded. All the included studies required patients provided written informed consent. Key words used to search for relevant publications included the following: (“aspirin” and “stroke”) or (“aspirin” and “prevention”).

### Data extraction

Titles and abstracts of the articles were screened by two reviewers (Hong Lei and Qian Gao) independently. Included articles for full text screening were compared during a consensus meeting. In case of disagreement, a third reviewer (Jian Xu) was consulted for the decision on inclusion or exclusion for full-text evaluation. Articles that did not contribute to the answer of our research questions after full text evaluation were excluded. After consensus the remaining articles were included for critical appraisal and assessed by two reviewers independently. Articles (RCT studies) were judged on scientific quality according to the CONSORT and STROBE statement (von Elm et al., 2007; Schulz et al., 2010).

### Data analysis and statistical methods

The significance of the combined odds ratio (OR) was determined by the *Z*-test, in which *P* < 0.05 was considered significant. The χ^2^-based Q statistical test was used for the assessment of the between-study heterogeneity, which was considered significant for *P* < 0.1. In analyses, if the heterogeneity was low, then we used a fixed-effect model, or else applied the random-effect model. Software of Review Manager 5.3 was used to perform the meta-analyses (available from Cochrane). When the heterogeneity was high, we collected sufficient information to conduct particular subgroup analyses to determine the population-aspirin interaction and stroke types-aspirin interaction. As the number of trials was small (≤10), a funnel plot was not used to assess publication bias.

## Results

**Figure 1:**
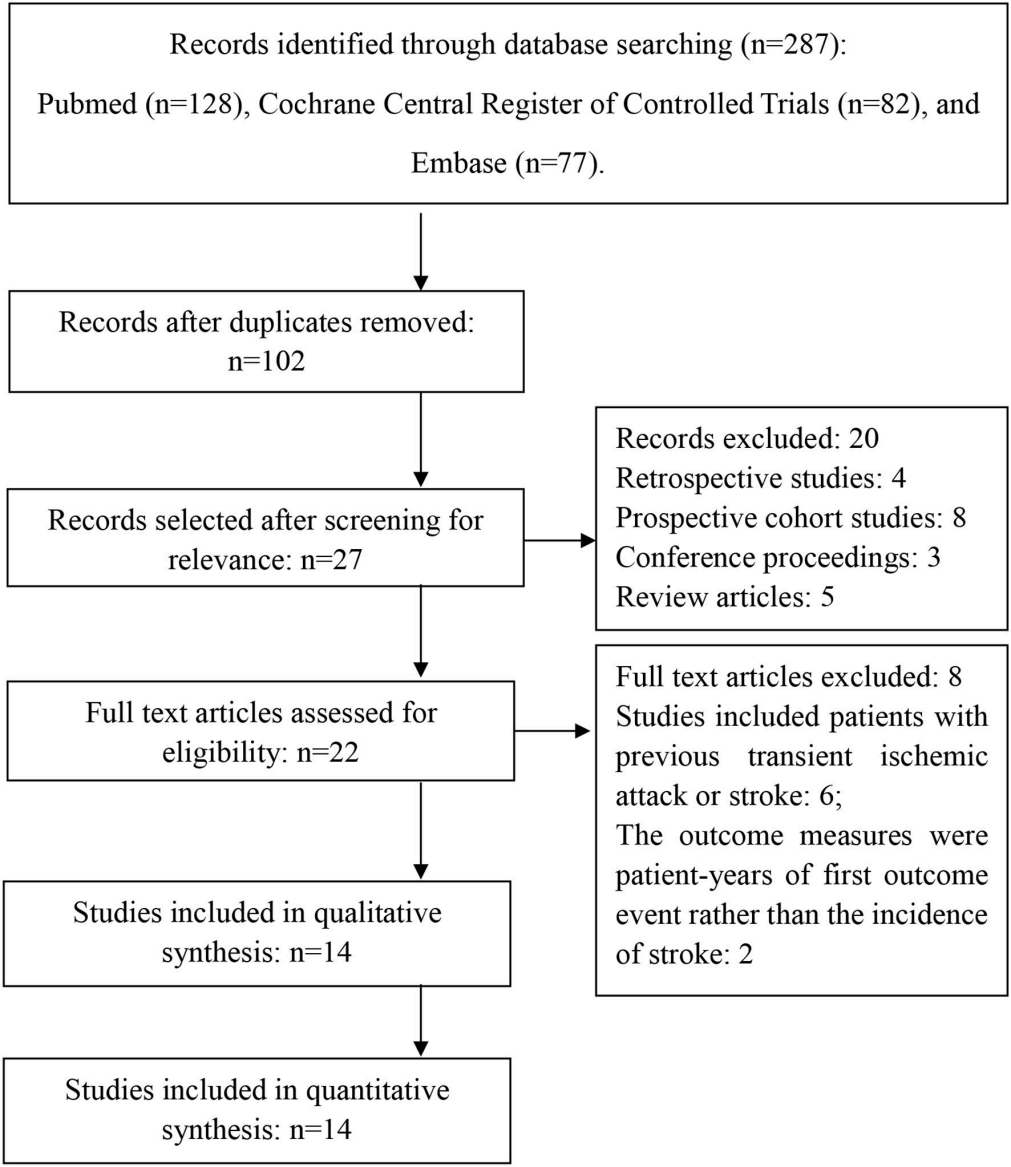
**Flow diagram of selected studies**.

Figure 1 shows the selective process after the search: Of the 14 included articles in this systematic review, a total of 8 studies enrolled patients with previous cardiovascular events and 7 studies enrolled volunteers with or without increased risk for cardiovascular diseases (Peto et al., 1988; Steering Committee of the Physicians' Health Study Research Group, 1989; Lindblad et al., 1993; Hansson et al., 1998; The Medical Research Council's General Practice Research Framework, 1998; Cook et al., 2000; Meade and Brennan, 2000; de Gaetano, 2001; Sacco et al., 2003; Cleland et al., 2004; Ridker et al., 2005; Ogawa et al., 2008; Fowkes et al., 2010; Kurth et al., 2011). The risk factors included: old age (≥65 years), hypertension, hypercholesterolemia, obesity, diabetes, and family history of premature myocardial infarction.

### Characteristics of the studies

The characteristics of the included articles are reported in Table 1 (see below).

**Table 1:** **Characteristics of 14 studies included in the review**.

**Study**	**Studied population**	**Participants (n)**	**Control (n)**	**Ischemic stroke (Participants/Control)**	**Severe adverse events**	
					**Hemorrhagic stroke (Participants/Control)**	**Gastrointestinal bleeds (Participants/Control)**
Ogawa et al. (2008)^8^	Patients with diabetes	81–100 mg aspirin daily (1262)	No aspirin (1277)	23/29	5/3	4/0
Ridker et al. (2005)^13^	Women	100 mg aspirin on alternate days (19,934)	100 mg placebo on alternate days (19,942)	170/221	51/41	127/91
Meade and Brennan (2000)^7^	Men with increased risk of coronary heart disease	75 mg aspirin daily (8105)	75 mg placebo daily (8071)	NA	NA	NA
Fowkes et al. (2010)^14^	Elderly people (50–75 years)	100 mg aspirin daily (1675)	100 mg placebo daily (1675)	44/50	5/5	9/8
Sacco et al. (2003)^15^	Patients with diabetes; Patients without diabetes	100 mg aspirin daily (519); 100 mg aspirin daily (1875);	No aspirin (512), No aspirin (1893)	NA	NA	NA
Cook et al. (2000)^16^	Elderly people (40–84 years)	325 mg aspirin on alternate days (11,010)	325 mg placebo on alternate days (3849)	110/42	NA	NA
Kurth et al. (2011)^17^	Women (≥45 years)	100 mg aspirin on alternate days (19,869)	100 mg placebo on alternate days (19,888)	170/221	51/41	NA
Peto et al. (1988)^18^	Male doctors	500 mg aspirin daily (3429)	No aspirin (1710)	NA	1/0	NA
Lindblad et al. (1993)^19^	Patients undergoing carotid endarterectomy	75 mg aspirin daily (117)	75 mg placebo daily (115)	NA	NA	NA
Cleland et al. (2004)^20^	Patients with heart failure	300 mg aspirin daily (91)	No aspirin (89)	NA	NA	NA
Hansson et al. (1998)^21^	Patients with increased blood pressure	75 mg aspirin daily (9399)	75 mg placebo daily (9391)	NA	NA	NA
The Medical Research Council's General Practice Research Framework (1998)^22^	Patients with increased risk for ischemic heart disease	75 mg aspirin daily (1268)	75 mg placebo daily (1272)	1/3	2/0	6/2
de Gaetano (2001)^23^	Patients with increased risk for cardiovascular disease	100 mg aspirin daily (2226)	No aspirin (2269)	NA	2/0	NA
Steering Committee of the Physicians' Health Study Research Group (1989)^9^	Healthy male physicians	325 mg aspirin on alternate days (11,037)	325 mg placebo on alternate days (11,034)	91/82	23/12	NA

A summary of study quality indicators is presented in Table 2. Randomization occurred in all 14 studies, but only 5 studies described the process of random sequence generation (Peto et al., 1988; The Medical Research Council's General Practice Research Framework, 1998; de Gaetano, 2001; Sacco et al., 2003; Ogawa et al., 2008). The use of allocation concealment was clearly stated only in three of the trials (The Medical Research Council's General Practice Research Framework, 1998; Cook et al., 2000; de Gaetano, 2001). All studies had a Jadad score of three or greater except one. Five trials were open-labeled (Peto et al., 1988; de Gaetano, 2001; Sacco et al., 2003; Cleland et al., 2004; Ogawa et al., 2008). Loss to follow-up was accounted for in all trials. None of the trials appeared to have substantial baseline differences between patients allocated to aspirin therapy vs. the comparator-arm.

**Table 2:** **Summary of quality indicators for studies assessing aspirin for the primary prevention of stroke**.

**Study, Year**	**Random sequence generation**	**Allocation concealment**	**Blinding of participants and outcome-assessors**	**Placebo-controlled**	**Lost to follow up accounted**	**Potential baseline difference**	**JADAD score (Range 0–5)**
Ogawa et al. (2008)^8^	Yes	No	No	No	Yes	No	3
Ridker et al. (2005)^13^	No	No	Yes	Yes	Yes	No	4
Meade and Brennan (2000)^7^	No	No	Yes	Yes	Yes	No	4
Fowkes et al. (2010)^14^	No	No	Yes	Yes	Yes	No	4
Sacco et al. (2003)^15^	Yes	No	No	No	Yes	No	3
Cook et al. (2000)^16^	No	Yes	Yes	Yes	Yes	No	4
Kurth et al. (2011)^17^	No	No	Yes	Yes	Yes	No	4
Peto et al. (1988)^18^	Yes	No	No	No	Yes	No	3
Lindblad et al. (1993)^19^	No	No	Yes	Yes	Yes	No	4
Cleland et al. (2004)^20^	No	No	No	No	Yes	No	2
Hansson et al. (1998)^21^	No	No	Yes	Yes	Yes	No	4
The Medical Research Council's General Practice Research Framework (1998)^22^	Yes	Yes	Yes	Yes	Yes	No	5
de Gaetano (2001)^23^	Yes	Yes	No	No	Yes	No	4
Steering Committee of the Physicians' Health Study Research Group (1989)^9^	No	No	Yes	Yes	Yes	No	4

### Primary prevention of all stroke and ischemic stroke with aspirin

**Figure 2:**
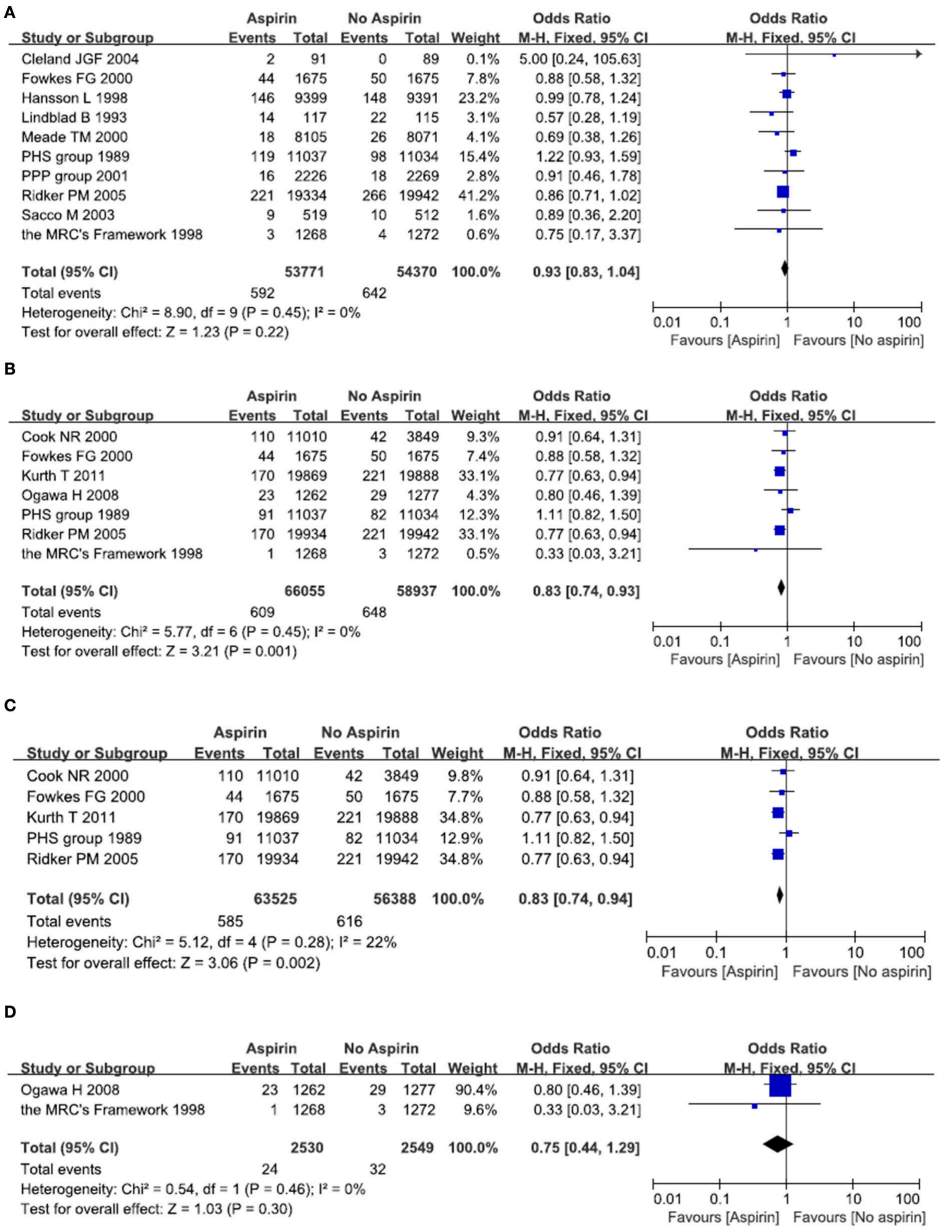
**Primary prevention of all stroke (A)** and ischemic stroke **(B–D)** with aspirin. Results were presented for all individuals combined **(B)**, apparently healthy individuals **(C)** and individuals without cardiovascular diseases **(D)**.

A total of 10 studies reported on the efficacy of aspirin in primary prevention of all strokes in people with or without cardiovascular risk factors (Steering Committee of the Physicians' Health Study Research Group, 1989; Lindblad et al., 1993; Hansson et al., 1998; The Medical Research Council's General Practice Research Framework, 1998; Meade and Brennan, 2000; de Gaetano, 2001; Sacco et al., 2003; Cleland et al., 2004; Ridker et al., 2005; Fowkes et al., 2010). When compared with those not taking aspirin, all included studies didn't show any difference in the incidence of stroke in people taking aspirin. Meta-analysis of these 10 studies resulted in a pooled OR of 0.93, 95% CI 0.83–1.04, indicating no benefit of aspirin in primary prevention of stroke of any kind (Figure 2A).

A total of seven studies reported on the efficacy of aspirin in primary prevention of ischemic stroke (Steering Committee of the Physicians' Health Study Research Group, 1989; The Medical Research Council's General Practice Research Framework, 1998; Cook et al., 2000; Ridker et al., 2005; Ogawa et al., 2008; Fowkes et al., 2010; Kurth et al., 2011). When compared with person not taking aspirin, two large randomized clinical trials demonstrated a decreased risk of stroke in people taking aspirin (Ridker et al., 2005; Kurth et al., 2011). Another five studies didn't show any difference in the incidence of ischemic stroke (Steering Committee of the Physicians' Health Study Research Group, 1989; The Medical Research Council's General Practice Research Framework, 1998; Cook et al., 2000; Ogawa et al., 2008; Fowkes et al., 2010). Meta-analysis of these seven studies resulted in a pooled OR of 0.83, 95% CI 0.74–0.93, indicating a mild but significant reduction in the incidence of ischemic stroke in those taking aspirin (Figure 2B).

Since aspirin may have a differential effect on different population, we therefore examined two subgroups: people without cardiovascular diseases; people with cardiovascular diseases such as diabetes and hypertension. A total of five studies reported on the efficacy of aspirin in primary prevention of ischemic stroke among people without cardiovascular diseases (Steering Committee of the Physicians' Health Study Research Group, 1989; Cook et al., 2000; Ridker et al., 2005; Fowkes et al., 2010; Kurth et al., 2011). Two large randomized clinical trials demonstrated a decreased risk of ischemic stroke in people taking aspirin (Ridker et al., 2005; Kurth et al., 2011), while another three studies didn't show any difference in the incidence of ischemic stroke (Steering Committee of the Physicians' Health Study Research Group, 1989; Cook et al., 2000; Fowkes et al., 2010). Meta-analysis of these five studies resulted in a pooled OR of 0.83, 95% CI 0.74–0.94, indicating a mild but significant reduction in the incidence of ischemic stroke in those relatively healthy person taking aspirin (Figure 2C). Another two studies demonstrated the effect of aspirin on people with cardiovascular diseases (The Medical Research Council's General Practice Research Framework, 1998; Ogawa et al., 2008). Meta-analysis of these two studies resulted in a pooled OR of 0.75, 95% CI 0.44–1.29, indicating no benefit of aspirin in prevention of ischemic stroke in people with cardiovascular diseases (Figure 2D).

### The risk of hemorrhagic stroke after exposure to aspirin

**Figure 3:**
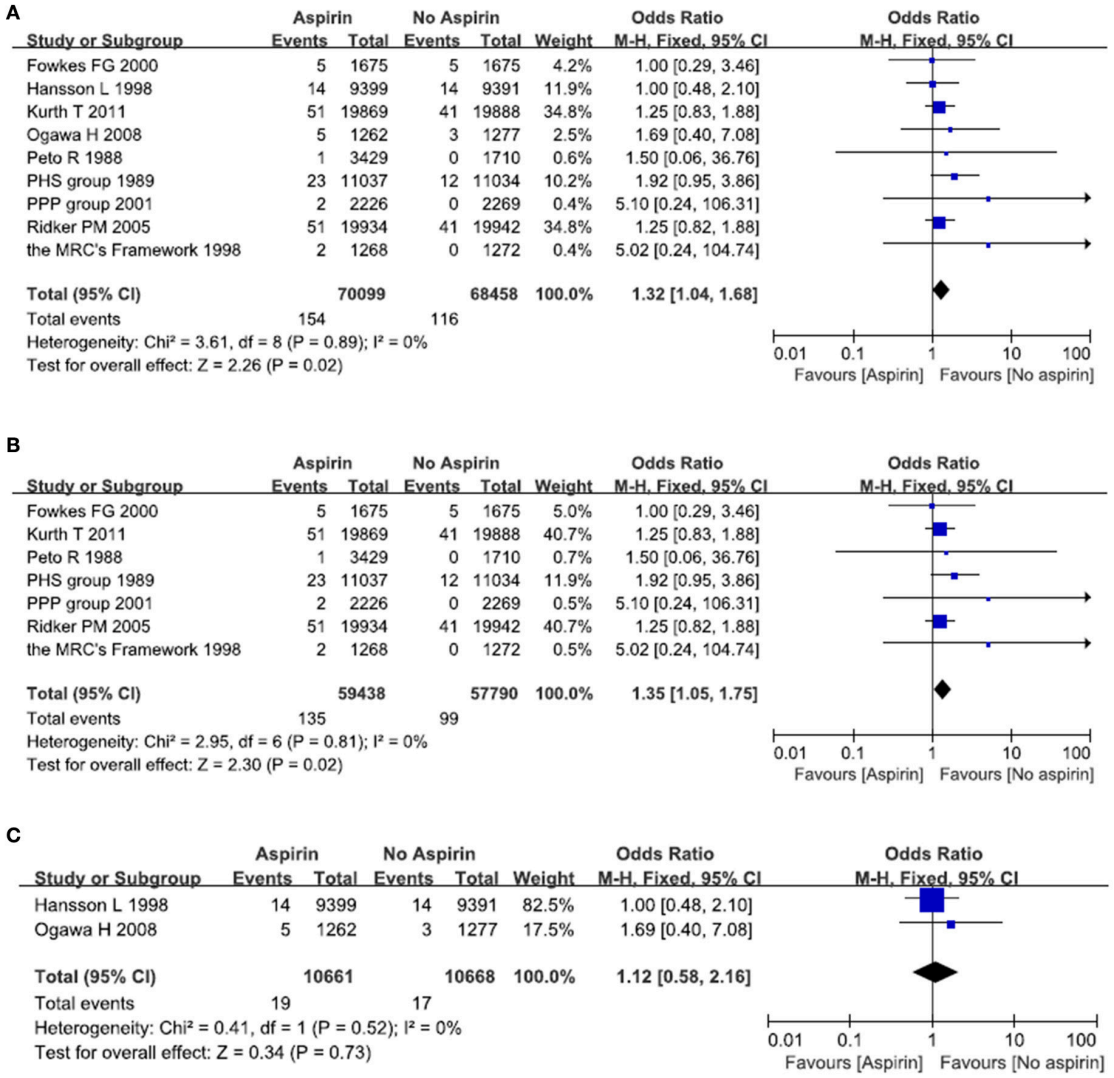
**The risk of hemorrhagic stroke after treatment with aspirin**. Forrest plot of fixed effect meta-analysis for pooled ORs of hemorrhagic stroke. Results were presented for all individuals combined **(A)**, apparently healthy individuals **(B)**, and individuals without cardiovascular diseases **(C)**.

A total of nine studies reported on the incidence of hemorrhagic stroke after exposure to aspirin (Peto et al., 1988; Steering Committee of the Physicians' Health Study Research Group, 1989; Hansson et al., 1998; The Medical Research Council's General Practice Research Framework, 1998; de Gaetano, 2001; Ridker et al., 2005; Ogawa et al., 2008; Fowkes et al., 2010; Kurth et al., 2011). There were 154 episodes of hemorrhagic stroke in the aspirin group, as compared with 116 in the placebo (no aspirin) group. Meta-analysis of these nine studies resulted in a pooled OR of 1.32, 95% CI 1.04–1.68, indicating an increased risk of hemorrhagic stroke in people taking aspirin (Figure 3A).

There was some evidence that the value of aspirin might vary with blood pressure and serum glucose. We therefore examined two subgroups: people without cardiovascular diseases; people with cardiovascular diseases such as diabetes and hypertension. In relatively healthy population, there is an increased risk of hemorrhagic stroke in the aspirin group as compared with the placebo (no aspirin) group. The pooled OR was 1.35 (95% CI 1.05–1.75) (Figure 3B). In patients with cardiovascular diseases, there was no significant difference between aspirin and placebo (no aspirin) for the risk of hemorrhagic stroke. The pooled OR was 1.12 (95% CI 0.58–2.16) (Figure 3C).

### The risk of major gastrointestinal bleeds after exposure to aspirin

**Figure 4:**
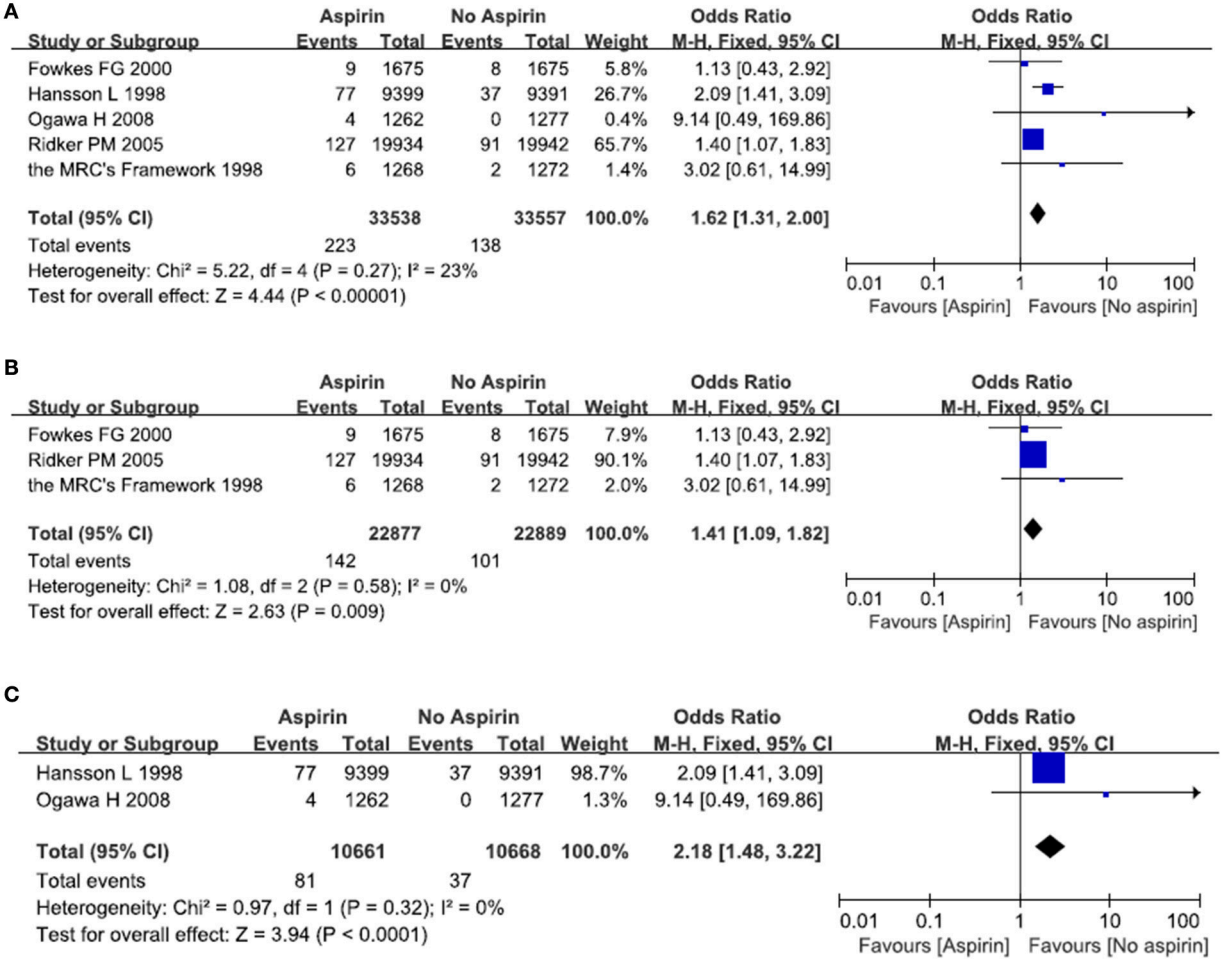
**The risk of severe gastrointestinal bleeds after treatment with aspirin**. Forrest plot of fixed effect meta-analysis for pooled ORs of serious gastrointestinal bleeds. Results were presented for all individuals combined **(A)**, apparently healthy individuals **(B)**, and individuals without cardiovascular diseases **(C)**.

A total of five studies reported on the incidence of major gastrointestinal bleeds after exposure to aspirin (The Medical Research Council's General Practice Research Framework, 1998; Hansson et al., 1998; Ridker et al., 2005; Ogawa et al., 2008; Fowkes et al., 2010). There were 223 episodes of major gastrointestinal bleeds in the aspirin group, as compared with 138 in the placebo (no aspirin) group. Meta-analysis of these five studies resulted in a pooled OR of 1.62, 95% CI 1.31–2.00, indicating an increased risk of major gastrointestinal bleeds in people taking aspirin (Figure 4A).

We then examined whether patients with cardiovascular diseases were at a greater risk of developing major gastrointestinal bleeds. In relatively healthy population, there is an increased risk of major gastrointestinal bleeds in the aspirin group as compared with the placebo (no aspirin) group. The pooled OR was 1.41 (95% CI 1.09–1.82) (Figure 4B). In patients with cardiovascular diseases, there was a significantly increased risk of major gastrointestinal bleeds in the aspirin group as compared with the placebo group (no aspirin). The pooled OR was 2.18 (95% CI 1.48–3.22) (Figure 4C) (see below).

## Discussion

When evaluating the effects of aspirin on ischemic stroke and hemorrhagic stroke respectively, our results indicated a significant 17 percent reduction in the risk of ischemic stroke and a significant 32 percent increase in the risk of hemorrhagic stroke, which suggested a net increase in the risk of hemorrhagic stroke in the study populations. This finding was particularly relevant, since under most circumstances, aspirin was prescribed to prevent ischemic cardiovascular events while actually the prophylactic use of aspirin might only provide limited protection against ischemic stroke. The harm from cerebral bleeds exceeded the benefit among those taking aspirin.

Numerous studies suggested the hypothesis that low-dose aspirin might be less effective in patients with cardiovascular diseases as compared with the general population. Although heterogeneity of trial results was low (*I*^2^ = 0%), we divided the studied populations into two groups: those with cardiovascular disease (such as diabetes, dyslipidemia, and hypertension) and those with not. Our results demonstrated that the positive effects of aspirin remained significant in the general population while patients with previous cardiovascular events seemed to derive little benefit from aspirin. Several mechanisms have been suggested that can be responsible for these findings. As for diabetes, it has been suggested that the involvement of aspirin insensitive Cox-2, as an additional source of TxA2, contributed to aspirin resistance (Halushka and Halushka, 2002). Thus, in patients with diabetes, platelets could be activated through different mechanisms that can lead to thrombosis despite aspirin therapy. As for hypercholesterolemia, a lower effect of aspirin in the presence of elevated values of total cholesterol was described in the Physician's Healthy Study and the Thrombosis Prevention Trial (Steering Committee of the Physicians' Health Study Research Group, 1989; The Medical Research Council's General Practice Research Framework, 1998). It has been shown that the increasing level of cholesterol was associated with reduced responsiveness of platelets to aspirin (Friend et al., 2003). As for hypertension, it has often been considered a contradiction to aspirin because of the concern that possible benefits in the prevention of occlusive events may be counterbalanced by an increased risk of cerebral bleeding (Hansson et al., 1998).

For patients who would experience severe adverse events such as hemorrhagic stroke after prophylaxis use of aspirin, aspirin was more likely to do harm than good. To our knowledge, severe adverse events related to aspirin included hemorrhagic stroke and major bleeds. The latter included serious bleeding from any tissue or organ. The most common one was gastrointestinal bleeding. Usually, patients who had serious gastrointestinal bleeding needed transfusion. Thus, in our study, we compared the risk of hemorrhagic stroke and severe gastrointestinal bleeds between the aspirin and no aspirin groups. Overall, these side effects were more common in the aspirin group than in the non-aspirin group. We then conducted particular subgroup analyses to determine whether aspirin could both increase the risk of serious adverse events in two different populations. Our findings showed that in the general population, aspirin increased the risk of specific adverse events such as hemorrhagic stroke and severe gastrointestinal bleeding. While in patients with cardiovascular diseases, aspirin use was only associated with a significant increase in the risk of severe gastrointestinal bleeding. It had no significant effect on hemorrhagic stroke.

These findings indicated that the benefit of aspirin was offset by the risk of bleeding in general population. In patients with cardiovascular diseases, aspirin failed to decrease the incidence of ischemic stroke, instead, it increased the risk of severe gastrointestinal bleeding. To our knowledge, Self-medication with aspirin is widespread, especially by many for whom there is increased risk of developing cardiovascular diseases. If our results are correct, patients with cardiovascular diseases will derive little protective benefit from aspirin. They will, however, be exposed to the risk of troublesome and serious bleeding. There might be no good for recommending aspirin use for apparently healthy person as well. Physicians should not be afraid to do nothing when there is no evidence that treatment is effective and clear evidence that it has side effects.

We acknowledge several limitations of our study. Firstly, we were only able to incorporate a total of three trials in our analyses to study the benefit and safety of aspirin in patients with cardiovascular diseases. Although there was no heterogeneity between studies, the small number of included trials reduced the statistical power. Thus, these findings must be interpreted with great caution. More randomized clinical trials for specific populations were needed to illustrate the benefit and harm of aspirin in the primary prevention of ischemic stroke among patients with cardiovascular diseases. Secondly, not all the included studies were double-blinded, randomized, controlled trials, a total of five studies were open, randomized clinical trials. Finally, most of the patients from the included studies were of Caucasian descent, suggesting a limited confidence when applying this data to other populations.

## Conclusions

This meta-analysis of randomized trials indicated that both the apparently healthy adults and patients with cardiovascular diseases will derive little protective benefit from aspirin considering the increased risk of severe bleeding events.

## Author contributions

HL and QG analyzed the data and wrote the manuscript. SL collected the data and performed the analyses. JX designed the study and amended the paper.

### Conflict of interest statement

The authors declare that the research was conducted in the absence of any commercial or financial relationships that could be construed as a potential conflict of interest.
